# An Optical MEMS Acoustic Sensor Based on Grating Interferometer

**DOI:** 10.3390/s19071503

**Published:** 2019-03-28

**Authors:** Mengying Zhang, Gaomi Wu, Dipeng Ren, Ran Gao, Zhi-Mei Qi, Xingdong Liang

**Affiliations:** 1 State Key Laboratory of Transducer Technology, Institute of Electronics, Chinese Academy of Sciences, Beijing 100190, China; zhangmengying411@sina.com (M.Z.); gaomi_wu@126.com (G.W.); rendipeng16@mails.ucas.ac.cn (D.R.); rgao@mail.ie.ac.cn (R.G.); 2 Science and Technology on Microwave Imaging Laboratory, Institute of Electronics, Chinese Academy of Sciences, Beijing 100190, China; xdliang@mail.ie.ac.cn; 3 School of Electronic, Electrical and Communication Engineering, University of Chinese Academy of Sciences, Beijing 100049, China

**Keywords:** MEMS, acoustic sensors, grating, interferometry, stability

## Abstract

Acoustic detection is of great significance because of its wide applications. This paper reports a Micro-Electro-Mechanical System (MEMS) acoustic sensor based on grating interferometer. In the MEMS structure, a diaphragm and a micro-grating made up the interference cavity. A short-cavity structure was designed and fabricated to reduce the impact of temperature on the cavity length in order to improve its stability against environment temperature variations. Besides this, through holes were designed in the substrate of the grating to reduce the air damping of the short-cavity structure. A silicon diaphragm with a 16.919 µm deep cavity and 2.4 µm period grating were fabricated by an improved MEMS process. The fabricated sensor chip was packaged on a conditioning circuit with a laser diode and a photodetector for acoustic detection. The output voltage signal in response to an acoustic wave is of high quality. The sensitivity of the acoustic sensor is up to −15.14 dB re 1 V/Pa @ 1 kHz. The output signal of the high-stability acoustic sensor almost unchanged as the environment temperature ranged from 5 °C to 55 °C.

## 1. Introduction

Acoustic detection is of great significance because of its wide applications to environmental monitoring, health monitoring, mechanical fault diagnosis, and building defect examination. Compared with the conventional acoustic sensors based on electric methods, such as capacitance sensors and piezoresistive sensors, the optical ones are immune to electromagnetic interference (EMI), capable of multiplexing, and able to achieve high sensitivity with small size. Many types of fiber-optic acoustic sensors, based on fiber Bragg gratings [1,2], intensity modulation [3], and interferometers, had been reported. Among them, the interferometric sensors, such as Fabry–Pérot interferometers [4,5,6], Mach–Zehnder interferometers [7,8,9], and Sagnac interferometers [10], have achieved high sensitivity and low minimum detectable pressure. 

With the development of MEMS (Micro-Electro-Mechanical System) technology, optical acoustic sensors based on the MEMS structure have attracted much attention. First of all, the structures of the MEMS acoustic sensors are able to be designed flexibly for specific requirements or high performance. Vibration films with different microstructures have been developed, replacing the common uniform flat ones to improve the sensitivity and stability of sensors [11,12]. In addition, with high-precision MEMS technology, micro-sized designs of sensors are able to be well realized [13,14]. Compared with sensors which only use the MEMS component as a part of the whole assembled structure, those with an integrated MEMS structure based on a grating interferometer show enhanced stability because their interference cavity is stable and precisely controlled. Besides this, the performance of the sensor is improved by taking advantage of the diffraction characteristics of the grating [15]. As there are still electro components in the common systems of the sensors based on grating interferometers, they are opto-electronic sensors. Although the sensors are not immune to EMI, they still have the advantages of high sensitivity and small size. In addition to acoustic signals [16], sensors based on grating interferometers have also been applied to detecting position [17], displacement [18], and acceleration [19]. 

Sensitivity and stability are both important performance parameters of acoustic sensors for application. Low stability against environmental temperature variations is a serious problem for optical acoustic sensors, especially for the highly sensitive interferometric ones. Most of the methods which have been reported to solve the problem concern the laser source or the signal processing system [20,21] with additional complex operation, devices, and cost. Some other research improved the stability by altering the design of the interference cavity, such as by matching the materials [22,23] and optimizing the interference light field [24]. More effort is still needed to develop acoustic sensors with high sensitivity, high stability, and a simple system.

In this work, an optical MEMS acoustic sensor based on grating interferometer was designed and fabricated. The short-cavity structure was designed to improve its stability against environmental temperature variations. The sensor chip was packaged with the optical devices and a conditioning circuit for acoustic detection. Then, the response of the acoustic sensor to acoustic signals and temperature was tested. 

## 2. Materials and Methods

### 2.1. The acoustic sensor design

**Figure 1:**
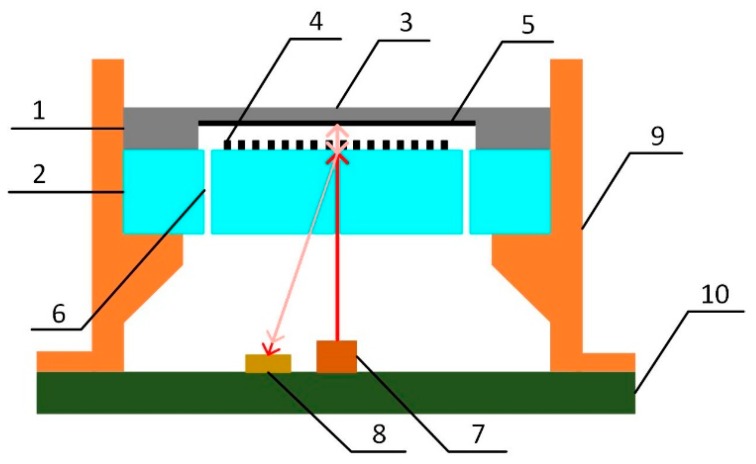
Schematic of the acoustic sensor. 1: Silicon substrate. 2: Glass substrate. 3: Diaphragm. 4: Grating. 5: Reflective layer. 6: Through hole. 7: Laser diode. 8: Photodetector. 9: Housing. 10: Printed circuit board.

The acoustic sensor reported in this work is shown in Figure 1. The MEMS sensor chip consists of two components: the diaphragm and the micro-grating. The vertical distance between the back surface of the diaphragm and the surface of the grating is the cavity length of the micro-grating interferometer, represented by *L*. 

A packaging structure was designed for the MEMS acoustic sensor chip. A laser diode with an integrated collimation structure was installed on the printed circuit board (PCB) to provide a 650 nm wavelength laser. It was placed vertically under the sensor chip and at the center. A photodetector matching with the laser was installed beside the laser diode at a specific distance. Both of the optical devices were connected with the circuit. 

When the acoustic sensor was operated, coherent light from the laser diode was vertically incident on the micro-grating. One part of the light was diffracted and reflected back, while another part passed through the grating and was vertically incident on the diaphragm. Then, the back surface of the diaphragm reflected the light back to the grating, and the light was diffracted again. The two diffracted light beams of the same order were at the same angle, and they interfered with the optical path difference of 2*L*. The intensity of the interference light at the first diffraction order of the grating is shown in equation (1) [15]:

(1) $$I_{1}=\frac{4I_{in}}{\pi^{2}}\sin^{2}(\frac{\phi }{2})=\frac{4I_{in}}{\pi^{2}}\sin^{2}(\frac{2\pi L}{\lambda })=\frac{2I_{in}}{\pi^{2}}[1-\cos(\frac{4\pi L}{\lambda })]$$

where *I*_in_ is the intensity of the incident light, *Φ* is the relative phase difference between the two diffracted light beams, and *λ* is the wavelength of the laser.

When acoustic pressure was loaded on the sensor, the diaphragm was deformed and the cavity length was changed. According to equation (1), the intensity of the interference light varied with the cavity length. Thus, the interference light was modulated by the acoustic wave. Then, it was detected by the photodetector, and the optical signal was processed by a conditioning circuit to output the voltage signal for acoustic detection. 

In order to enhance the sensitivity of the acoustic sensor, its structure was optimized. The diaphragm was designed with 3 μm thickness and 6 mm diameter. A reflective layer was added on the back surface of the diaphragm to enhance the reflection and improve the detection sensitivity. The micro-grating in the sensor decided the path of the light. Considering the structure of the sensor, a larger diffraction angle would separate the outgoing interference light well, with a shorter vertical distance between the grating and the photodetector. This reduced the diffusion of the outgoing light and avoided interference by light of other orders at the same time, which meant higher sensitivity and lower noise. According to the diffraction characteristic of the grating, with vertically incident light, the angles of the diffraction orders are decided by the grating period, and a smaller period means a larger angle. The designed grating period is 2.4 µm, and the angle of the first diffraction order is calculated as 15.714°. 

In order to improve the stability of the acoustic sensor against environment temperature variations, a short-cavity structure was designed. For interferometric sensors, the main reason for the stability problem is that the cavity length varies with the temperature. The relationship between them is shown in equation (2):

(2) $$dL=\alpha L_{0}dT$$

where α is the thermal expansion coefficient of the cavity. According to the equation, the shorter the cavity length is, the weaker the temperature influence is. The designed cavity length was reduced to 17 µm. In addition, to reduce the air damping—which became serious because of the small volume of the short cavity—through holes were made in the glass substrate to connect the cavity and the atmosphere.

### 2.2. The MEMS sensor chip fabrication

**Figure 2:**
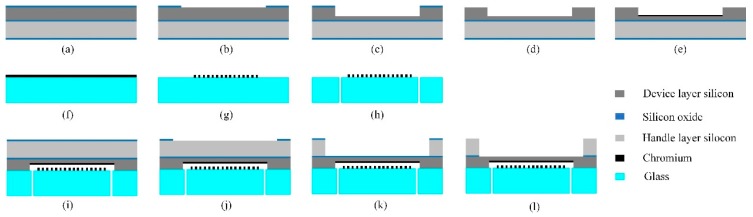
Micro-Electro-Mechanical System (MEMS) process of the acoustic sensor chip.

The MEMS process of the acoustic sensor chip was developed and optimized for the short-cavity grating interferometer structure, as Figure 2 shows. The diaphragm component was fabricated on a silicon-on-insulator (SOI) wafer, and the micro-grating was made on a borosilicate glass wafer.

(a) Silicon oxide films 500 nm in thickness were deposited on both surfaces of the SOI substrate by thermal oxidation.
(b) On the device layer, the silicon oxide film in the cavity area was etched by reactive ion etching (RIE).
(c) With the silicon oxide film as the mask, silicon in the device layer was etched by the wet method for the cavity.
(d) The remaining silicon oxide film on the device layer was removed by RIE.
(e) A 50 nm chromium film for the reflective layer was sputtered on the device layer and patterned.
(f) A 50 nm chromium film for the grating was sputtered on the glass substrate.
(g) The chromium film was patterned by ion beam etching (IBE).
(h) Through holes were made in the glass substrate by laser cutting.
(i) The two wafers were assembled by anodic bonding.
(j) On the handle layer of the SOI substrate, the silicon oxide film in the diaphragm area was etched by RIE.
(k) The silicon of the handle layer in the diaphragm area was etched by deep reactive ion etching (DRIE) until it reached the buried oxide layer.
(l) The exposed silicon oxide film was etched by RIE.

**Figure 3:**
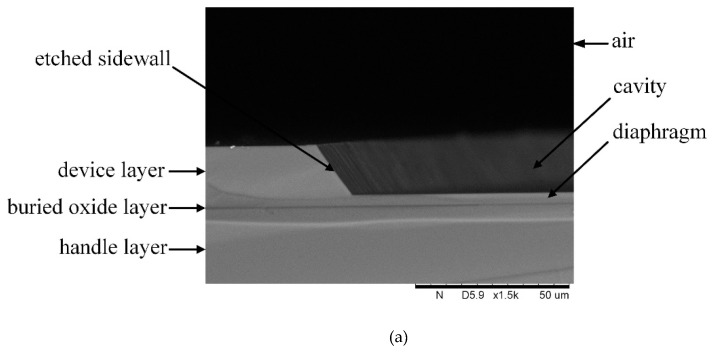

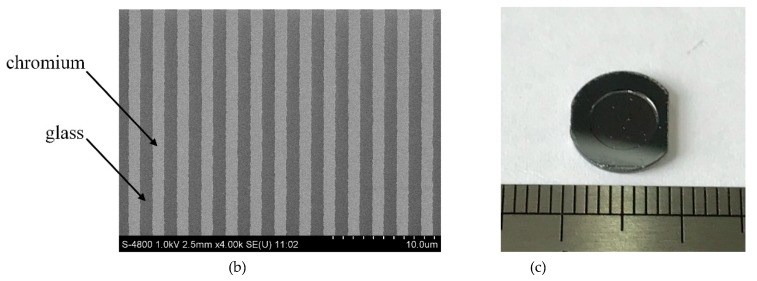
Photographs of the fabricated samples: (**a**) the etched cavity; (**b**) the micro grating; (**c**) the sensor chip.

As shown in Figure 3a, unlike the conventional process to fabricate a silicon diaphragm on an SOI substrate, an approximately 17 μm deep cavity was made in the 20 μm thick device layer and the remaining silicon in the device layer was for the diaphragm. Figure 3b shows that the 2.4 μm period grating in the sensor was fabricated with high precision. At last, the bonded wafer was divided into sensor chips with 10 mm diameter, as Figure 3c shows.

### 2.3. The Methods

The interference spectrum of the interference cavity was measured using an optical spectrum analyzer (YOKOGAWA, AQ6379B). Acoustic signals in the test of the sensor’s performance were generated from a speaker driven by a waveform generator (Agilent, 33500B). The output voltage signal from the sensor was detected by a mixed signal oscilloscope (Keysight, MSO-X 2024A). The frequency characteristic of the fabricated sensor was investigated by an acoustic sensor calibration system consisting of an input/output generator module (B&k, 3160-A-042), a phase calibrator (GRAS, 51AB), a calibration microphone (B&k, 4193-L-004), and measurement software (B&K, PULSE LabShop). In the stability test, the environmental temperature was controlled using a temperature and humidity test chamber.

## 3. Results and Discussion

### 3.1. The MEMS Sensor Chip

**Figure 4:**
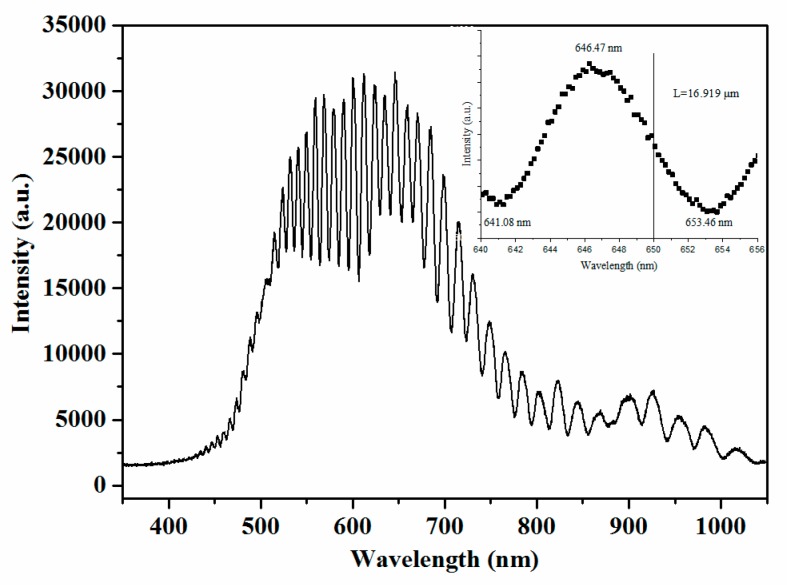
Interference spectrum measured for determining the cavity length.

The interference cavity length of the fabricated sensor chip was measured from its interference spectrum with broadband incident light, as Figure 4 shows. The cavity length is calculated as 16.919 µm. There was a small error between the fabricated cavity length and the designed one. As shown in the interference spectrum, the wavelength difference between the wave crest and the wave trough around 650 nm is 7 nm. It is indicated that the short-cavity structure is not quite sensitive to the wavelength of the laser. Given the problem that the laser diode is sensitive to variations in the environment, the short-cavity design is beneficial to improving the stability. In addition, calculated using Equation (2), the temperature coefficient of the cavity length is 0.0423 nm/°C. With a 650 nm laser source, the coefficient of the phase difference is 0.047°/°C. The temperature coefficient of the short cavity is so low that it is hardly affected by the environmental temperature. 

### 3.2. Response to Acoustic Signal

**Figure 5:**
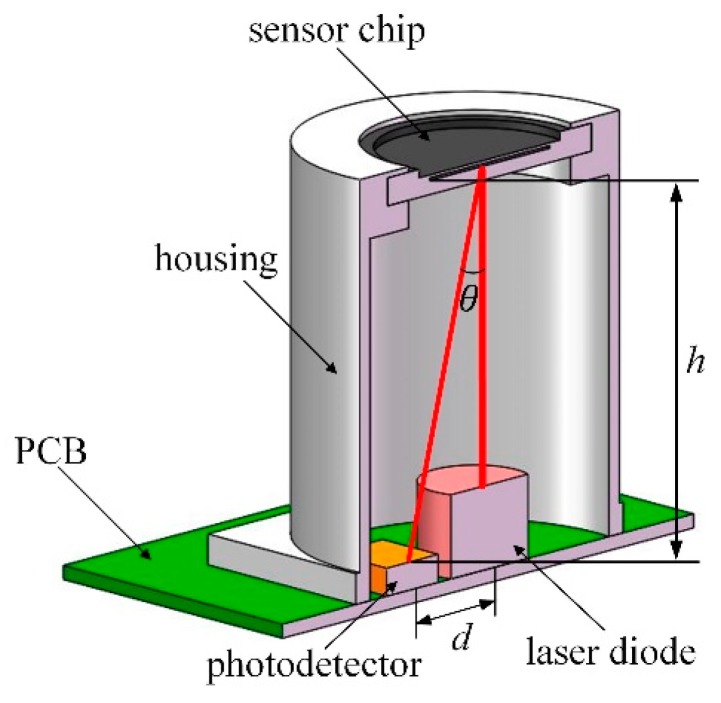
Schematic diagram of the packaged acoustic sensor.

The packaging structure of the acoustic sensor designed based on the optical interference principle is shown in Figure 5. The MEMS sensor chip was packaged on the top of the housing and the diaphragm was exposed in order to sense acoustic signals. For the light path, the height from the surface of the photodetector to the surface of the grating (*h*) and the distance between the center of the laser diode and the center of the photodetector (*d*) are decided by the angle of the grating’s first diffraction order (*θ*). This means that the height of the packaged sensor is mainly limited by the period of the grating, as enough space is needed for the optical devices. Both the housing and the optical devices were installed on the PCB with a conditioning circuit for acoustic detection. The circuit consisted of the drive part connected with the laser diode, the signal process part connected with the photodetector, and the filter part. Then, the output voltage signal from the circuit was used to detect acoustic signals.

**Figure 6:**
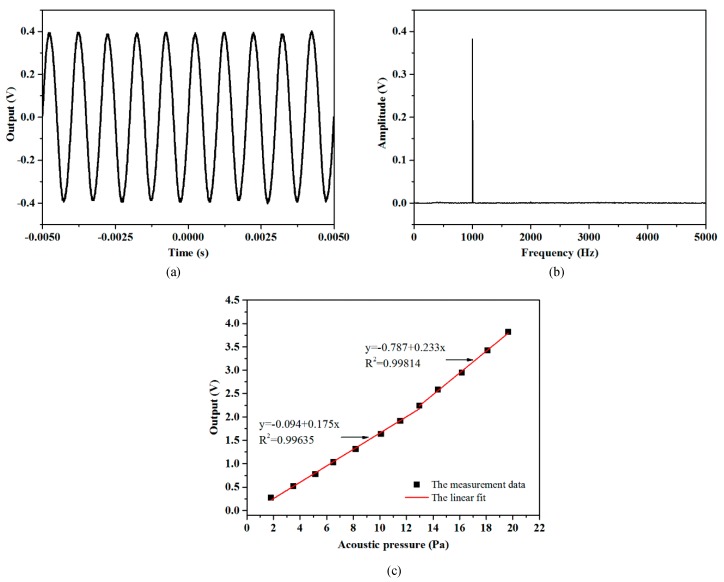
Response of the sensor measured with an acoustic signal of 1 kHz frequency: (**a**) time domain signal; (**b**) FFT of the time domain signal; (**c**) the relationship between the acoustic pressure and the sensor’s output.

The response of the sensor to an acoustic signal of 1 kHz frequency is shown in Figure 6. The waveform of the output voltage signal and its Fast Fourier Transformation (FFT) indicate that the output of the sensor for acoustic detection is of high quality. With calibration by the standard microphone, the output values of the sensor under different acoustic pressures are shown in Figure 6c. For acoustic pressure lower than 13 Pa, the response is linear and the sensitivity is −15.14 dB re 1 V/Pa, according to the linear fit with a 0.99635 R^2^ value. When the pressure becomes higher, the response increases. 

**Figure 7:**
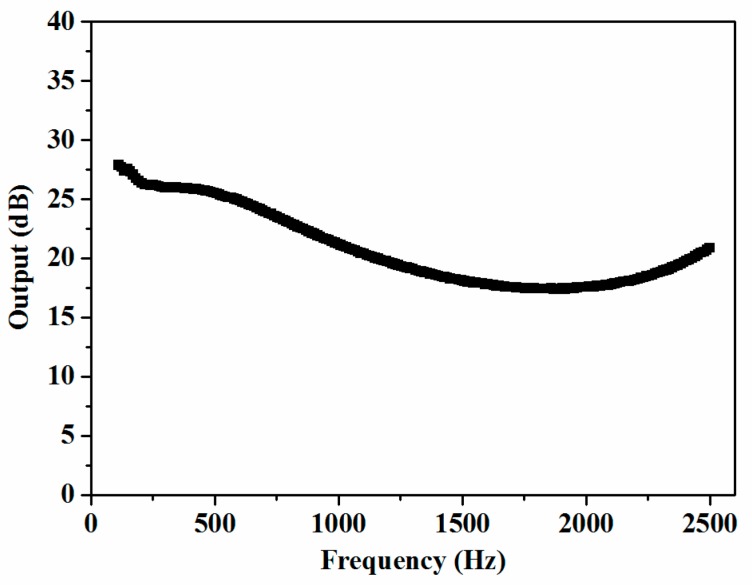
Frequency response of the fabricated sensor compared with that of the calibration microphone.

The frequency characteristic of the fabricated sensor was investigated by the acoustic sensor calibration system described in Section 2.3. When the system was operated, the generator drove the calibrator to provide a sweep acoustic signal. Then, the outputs of the sensor and the calibration microphone were imported into the input/output module and processed by the software. In the measurement software, the output of the sensor for every frequency was compared with that of the calibration microphone. Then, the difference between them was calculated; these differences are shown in Figure 7. The sensitivity of the calibration microphone is −37.8 dB re 1 V/Pa and the flatness of its frequency response was ±1 dB from 0.12 Hz to 7 kHz for the pressure field, according to its specifications. Defining the response of the calibration microphone as stable, the difference between the highest sensitivity and the lowest one of the fabricated sensor is 10 dB within the frequency range from 100 Hz to 2.5 kHz. 

### 3.3. Stability of the Sensor

**Figure 8:**
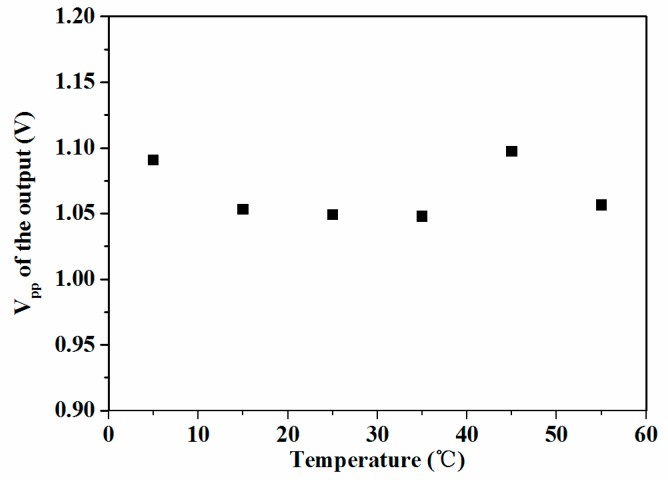
Response of the fabricated sensor with varying temperature.

The stability of the acoustic sensor against environment temperature variations was tested in the temperature and humidity test chamber. There was no additional design of the sensor chip or the circuit for freezing, so the sensor was not operated under 0 °C. Further, the operating temperature ranges of the laser diode (−36 °C to 65 °C) and the photodetector (−25 °C to 85 °C) in the sensor system limited the highest operating temperature. In the test, the environment temperature was controlled from 5 °C to 55 °C. The measured peak-to-peak values of the sensor’s response to the fixed acoustic signal are shown in Figure 8. The mean of the values is 1.065 V, and the changes are within 0.049 V, meaning 4.6%, when the temperature varied by 50 °C.It is indicated that the acoustic sensor with a designed short-cavity structure achieved high thermal stability.

## 4. Conclusions

In this work, an optical MEMS acoustic sensor based on grating interferometer was designed and fabricated. The structure of the sensor chip was optimized to enhance the acoustic detection sensitivity, and a short-cavity structure was designed to improve the stability against environment temperature variations. The MEMS process was developed to fabricate the sensor chip with a short-cavity design. The fabricated sensor chip was packaged with optical devices and a conditioning circuit for acoustic detection. The output voltage signal of the sensor is of high quality. The sensitivity of the acoustic sensor is up to −15.14 dB re 1 V/Pa @ 1 kHz, and the variation of the sensitivity is within 10 dB in the frequency range from 100 Hz to 2.5 kHz. The temperature coefficient of the short-cavity interferometer is very low, offering the sensor high thermal stability. The output signal of the developed acoustic sensor almost unchanged as the environment temperature varied from 5 °C to 55 °C.
